# Adapting Efficiency Analysis in Health Systems: A Scoping Review of Data Envelopment Analysis Applications During the COVID-19 Pandemic

**DOI:** 10.3390/jmahp12040024

**Published:** 2024-10-22

**Authors:** Athanasios Mitakos, Panagiotis Mpogiatzidis

**Affiliations:** Department of Midwifery, School of Health Sciences, University of Western Macedonia, 50200 Ptolemaida, Greece; dmw00017@uowm.gr

**Keywords:** healthcare efficiency, COVID-19, data envelopment analysis, health systems, scoping review, global health challenges

## Abstract

**Objective:** To synthesize the current evidence base concerning the application of Data Envelopment Analysis (DEA) in healthcare efficiency during the COVID-19 pandemic using a scoping review of 13 primary studies. **Methods:** We consulted databases including Web of Science (WoS) and Scopus, as well as manual search entries up to September 2022. Included studies were primary applications of DEA for assessing healthcare efficiency during the COVID-19 pandemic. Key findings derived from thematic analysis of repeating pattern observations were extracted and tabulated for further synthesis, taking into consideration the variations in DMU definitions, the inclusion of undesirable outputs, the influence of external factors, and the infusion of advanced technologies in DEA. **Results:** The review observed a diverse application of DMUs, ranging from healthcare supply chains to entire national health systems. There was an evident shift towards incorporating undesirable outputs, such as mortality rates, in the DEA models amidst the pandemic. The influence of external and non-discretionary factors became more pronounced in DEA applications, highlighting the interconnected nature of global health challenges. Notably, several studies integrated advanced computational methods, including machine learning, into traditional DEA, paving the way for enhanced analytical capabilities. **Conclusions:** DEA, as an efficiency analysis tool, has exhibited adaptability and evolution in its application in the context of the COVID-19 healthcare crisis. By recognizing the multifaceted challenges posed by the pandemic, DEA applications have grown more comprehensive, integrating broader societal and health outcomes. This review provides pivotal insights that can inform policy and healthcare strategies, underscoring the importance of dynamic and comprehensive efficiency analysis methodologies during global health emergencies.

## 1. Background and Introduction

The utilization of Data Envelopment Analysis (DEA) as a tool to appraise the efficiency of diverse entities, predominantly Decision Making Units (DMUs), has firmly entrenched itself within the annals of operations research and management science since its seminal introduction in the late 1970s by Charnes, Cooper, and Rhodes [1]. This non-parametric linear programming technique has been instrumental in offering insights into the relative efficiency of a conglomerate of DMUs that metamorphose multiple inputs into a myriad of outputs. Over successive decades, the ubiquity of DEA has permeated an expansive array of sectors, with the intricate realm of healthcare emerging as a focal point, attributed to the multifaceted and multi-dimensional intricacies inherent to health systems [2].

The advent of the COVID-19 pandemic precipitated an unparalleled perturbation within the global health milieu. Health infrastructures, traditionally appraised via established indicators encompassing service delivery, accessibility, and patient-centric satisfaction, found themselves ensnared within a maelstrom of unforeseen challenges [3]. As the pandemic’s magnitude augmented, the imperative to decipher the efficiency of these infrastructures in the face of this global crisis accentuated, underscoring the versatility and relevance of DEA within this altered framework.

Historically, the prowess of DEA has been its adeptness in accommodating a spectrum of DMUs, spanning microcosmic entities such as individual healthcare facilities to macroscopic structures like entire nations [4]. This adaptability assumed paramount importance within the COVID-19 milieu, with a plethora of research endeavors employing an eclectic array of DMU categorizations to ascertain the efficiency of healthcare frameworks. Concurrently, the pandemic ushered in innovative dynamics into DEA evaluations, epitomized by the necessity to incorporate previously marginalized undesirable outputs, such as mortality indices [5].

In tandem, the intricate nexus of exogenous and non-discretionary variables, ranging from geopolitical undercurrents to socio-economic stratifications, garnered amplified attention. In light of the pandemic’s pervasive impact, transcending geopolitical demarcations and permeating every stratum of global society, an astute comprehension of these omnipresent determinants emerged as quintessential for a comprehensive DEA appraisal [6].

In the contemporary era, characterized by the dawn of the Fourth Industrial Revolution, the amalgamation of time-honored DEA methodologies with emergent computational stratagems signifies a renaissance in efficiency diagnostics. Ranging from avant-garde data analytics to the assimilation of machine learning frameworks, the symbiosis of DEA with technological innovations has paved the way for novel academic inquiries and pragmatic implementations [7].

This scoping review, therefore, embarks on a mission to amalgamate the extant literature on DEA’s application within health infrastructures, with an accentuated emphasis on the COVID-19 pandemic’s backdrop. Through an assiduous scrutiny of germane academic contributions, this review aspires to illuminate the metamorphosing paradigms, spotlight predominant trajectories, and proffer a critical evaluation of DEA’s evolution within this fluid and perpetually transformative global health landscape.

## 2. Methodological Framework

This scoping review is anchored on the five-stage framework delineated by Arksey and O’Malley (2005, p. 22) [8]. Our meticulous approach is structured as follows:

### 2.1. Articulation of Research Questions

A dual-pronged inquiry is formulated to comprehend:

- Which are the intricate dynamics of healthcare efficiency amidst the COVID-19 pandemic across diverse settings?
- Which are the multifarious factors that might have influenced healthcare operational efficiency during this unparalleled health crisis?

### 2.2. Comprehensive Literature Exploration

Search terminologies were intricately crafted, categorically delineated into three thematic realms befitting the purview of this review. These encompassed health sector efficiency, contextual integration with the COVID-19 pandemic, and the geographical settings involved. A Boolean algorithm structured as: (“Healthcare” OR “Health Sector Efficiency”) AND (“COVID-19” OR “Pandemic”) AND (“Settings” OR “Geographical Variation”) refined our search precision.

Digital repositories explored included Web of Science (WoS) and Scopus. Additionally, to ensure no seminal works eluded our scrutiny, an assiduous manual exploration of bibliographies—referred to academically as “manual-searching”—was pursued post-electronic database examination.

For inclusionary precision, our parameters encompassed original research or systematic reviews, regardless of design, from any global locale focusing on health sector efficacy during the COVID-19 pandemic. Moreover, the temporal boundary was demarcated at studies published up until 30 September 2022.

Studies were filtered based on chronological relevance, focus on healthcare settings encountering the pandemic, and data on efficiency metrics pre and post pandemic onset. The realm of exclusion comprised non-English manuscripts, non-peer-reviewed content (inclusive of preprints, conference abstracts, and editorials), and redundant publications.

### 2.3. Diligent Study Assimilation

**Figure 1:**
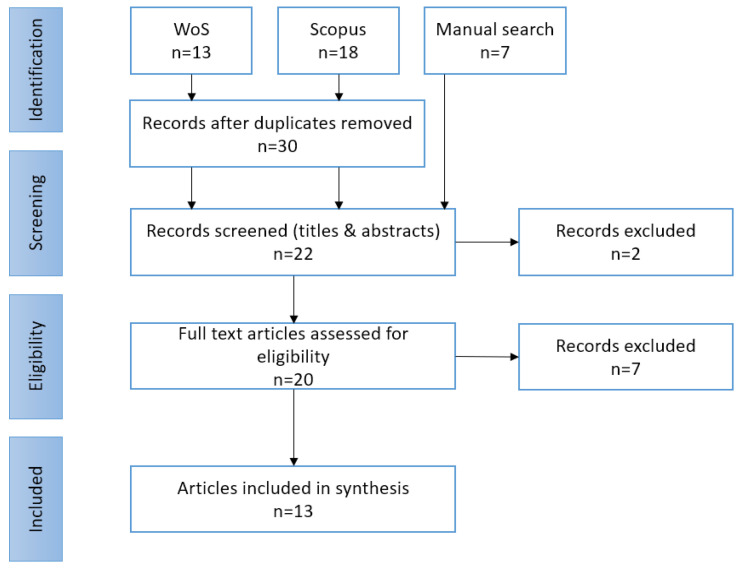
Search and inclusion process developed on the basis of the adopted methodology (PRISMA).

An iterative tri-phase selection protocol, predicated upon the PRISMA-ScR guidelines, was adopted. Initial screening involved titles, subsequently abstracts, culminating in an exhaustive examination of the complete manuscripts. The implementation of the adopted assimilation methodology resulted in a total of 13 scientific publications eligible for review (refer to Figure 1 for a schematic representation of the process).

### 2.4. Data Synthesis and Stratification

Each scholarly contribution was subjected to a detailed assessment employing a bifurcated approach as follows: quantitative delineation and overarching thematic dissection. Quantitatively, the ensuing facets were culled: Authorial Details, Key Methodology/Technique, Main Focus/Contribution, Evolution and Diversification, Temporal Dynamics, Selective Variation of DMU, Incorporation of Undesirable Outputs, Emphasis on External and Non-Discretionary Factors, and Integration of Modern Technologies. The thematic analysis unearthed profound insights, authorial pearls of wisdom, theoretical postulations, and pertinent revelations within the realm of DEA healthcare efficiency assessment amidst the COVID-19 pandemic (refer to Table 1 for a detailed tabular representation).

### 2.5. Data Analysis

The analysis of the collected data was structured around a thematic synthesis approach, allowing for the extraction and integration of findings from various studies. This method involved the following bifurcated analytical strategy: quantitatively capturing authorial details, methodologies, main focus, and key contributions of each study; and qualitatively through thematic analysis to identify patterns and insights within the data. This combination of quantitative and qualitative analysis provided a holistic understanding of the applied DEA methodologies and their evolution in response to the pandemic’s challenges.

### 2.6. Identification of Categories

The identification of thematic categories was guided by the recurring patterns and insights derived from the thematic analysis. Categories were established based on the variation in Decision Making Unit (DMU) definitions, the inclusion of undesirable outputs, the role of external and non-discretionary factors, and the integration of advanced technologies in DEA applications. Each category was developed to encapsulate a distinct aspect of DEA application within the healthcare context during the COVID-19 pandemic, reflecting both methodological diversifications and adaptations necessitated by the health crisis.

The categories were further validated by the alignment with established DEA literature and current trends in healthcare performance analysis during the pandemic. This approach not only grounded the review in contemporary scholarly discourse but also ensured that the categories reflected the actual complexities and nuances encountered in real-world applications of DEA during an unprecedented global health emergency.

For assurance of correct English language use, LLM grammar, syntax, and spelling correction were employed.

### 2.7. Results Compilation, Distillation, and Exposition

**Figure 2:**
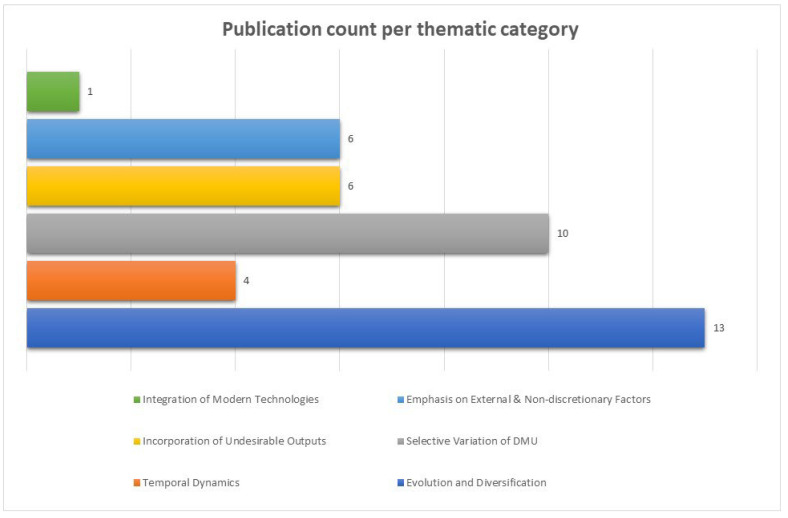
Bar chart depicting the count of articles per category of the thematic analysis. Each article may feature in more than one thematic categories, created by author.

Gleaned insights from every article underwent meticulous abstraction and were systematically enshrined within an organized matrix (refer to Figure 2 for a contextual graphical depiction).

Given the fact that a variety of publications exhibited more than one identified pattern, the thematic analysis categorization resulted in the same articles featuring in multiple categories (refer to Figure 2 for a detailed depiction of the article categorization based on the thematic analysis).

## 3. Results

### 3.1. Evolution and Diversification of DEA Methodologies in Health System Efficiency Analysis

In a meticulous exploration of the evolution and diversification of DEA methodologies in efficiency analysis as depicted in the works under study, a significant part of the seminal works reviewed came to the fore. Azadi et al. (2022) exemplify this progress with their groundbreaking introduction of a network RDM model tailored for healthcare supply chains during pandemics, a departure that underscores the adeptness in handling diversified data [9]. Concurrently, Breitenbach et al. (2021) utilize the VRS approach, deeply entrenched in microeconomics, to deliver an expansive efficiency evaluation, further extolling the versatility of DEA methodologies, particularly in treating undesirable outputs [10]. Subsequent works by Klumpp et al. (2022) and Kuzior et al. (2022), respectively, emphasize the nuances of the classic DEA model and the adoption of multivariate exploratory techniques [11,12], illuminating the realm of efficiency analysis with refined methodologies and sophisticated analytical tools. The holistic approach of Lupu and Tiganasu (2022), encapsulating various data sources and determinant categories [13], sets a precedent for dynamic efficiency evaluations, whereas the innovative strategies of Mourad et al. (2021) merge nonparametric mathematical programming with Tobit regression [14], championing a multi-model analytical paradigm.

As the trajectory of DEA methodologies continues to ascend, Ordu et al. (2021) artfully enhance traditional methodologies by integrating both CCR and BCC models [15], further rectifying ranking limitations through the super efficiency DEA method. In a similar vein, Pereira et al. (2022) introduce a riveting diversification by fusing multi-stage systems with simulations, accentuating the delicate balance between social welfare and resource optimization [16]. Adabavazeh et al. (2020) usher in a harmonized analytical paradigm, intertwining diverse evaluation indices and underlining the pivotal BCC output-based model [17]. Pioneering contributions, such as those by Mariano et al. (2021), leverage the NDEA to discern regional disparities [18], whereas Md Hamzah, Yu, and See (2021) introduce a trailblazing three-stage NDEA model [19], epitomizing the intricate dynamics of resource utilization. Furthermore, the synergy between traditional DEA approaches and the ‘Maximal Balance Index’ as demonstrated by Mohanta et al. (2021) provides a lucid insight into India’s pandemic management prowess [20]. Finally, the audacious merger of DEA and machine learning by Xu et al. (2021) heralds a new era in efficiency analysis [21], highlighting the symbiotic relationship between conventional analyses and contemporary predictive techniques, thus offering profound insights into the complex interplay of efficiency scores and their determinants.

Reflecting upon this anthology of articles, both the CCR and BCC models emerge as DEA stalwarts. The symbiotic integration of avant-garde techniques like Neural Networks and Decision Trees indeed hints at a dynamic methodological evolution.

### 3.2. Temporal Dynamics and Methodological Syntheses in DEA Health System Efficiency Analysis

At the confluence of temporal intricacies and methodological advancements lies a compelling narrative of efficiency analysis across various scientific explorations, especially as observed in the realm of COVID-19 research. A multitude of studies embrace diverse temporal datasets, ranging from succinct cross-sectional snapshots like that of Ordu et al. (2021) and Pereira et al. (2022) [15,16], to more exhaustive temporal intervals as evidenced in Klumpp et al.’s weekly segmented data [11] (2022) and Xu et al.’s expansive year-long assessment (2021) [21]. These endeavors adopt a plethora of analytical methodologies to delve into efficiency; notably, the Data Envelopment Analysis (DEA) emerges as a predominant tool, being wielded with unique specifications across various studies. For instance, while Klumpp et al. (2022) innovatively incorporated time-series efficiency via DEA window analysis [11], Mourad et al. (2021) adjusted the model orientation based on variable selection [14], and Mohanta et al. (2021) introduced the ‘Maximal Balance Index’ for an all-encompassing DMU ranking [20]. Moreover, there is an evident synergy of DEA with other paradigms, whether its Mariano et al.’s (2021) fusion with Network DEA to delineate regional disparities [18], or Xu et al.’s (2021) blend of DEA and machine learning to enhance predictive acuity [21]. This scoping review, thus, captures the rich tapestry of methodological innovations superimposed on varied temporal canvases, underscoring the domain’s dynamic evolution in deciphering efficiency amid global health crises.

### 3.3. Selective Variation of DMU Definition in DEA Health System Efficiency Analysis

In the expansive field of DEA Efficiency Analysis, Decision Making Units (DMUs) are pivotal entities that serve as units of assessment. Analyzing the collected articles, a diverse array of DMU definitions surfaces, unified by their overarching focus on healthcare systems in response to the COVID-19 pandemic. The selective variation is palpable. Azadi et al. (2022) delve into the healthcare supply chains, introducing a model that engages directly with ratio data [9]. Breitenbach et al. (2021) spotlight the healthcare system of each country, leveraging the variable returns to scale (VRS) approach, with an emphasis on input minimization concerning undesirable outputs [10]. Klumpp et al. (2022) pivot toward an output-oriented ratio form anchored in constant returns to scale (CRS) for countries’ pandemic responses [11]. Subsequent articles delineate DMUs ranging from global healthcare system models, European nations, and healthcare systems of densely populated countries to more nuanced entities like hospitals and universities. For instance, Kuzior et al. (2022) categorize countries based on healthcare system models [12], while Ordu et al. (2021) adopt a super efficiency DEA method assessing entities like universities and hospitals [15]. Further explorations by Pereira et al. (2022) and Adabavazehet al. (2020) focus on country-wide pandemic responses and health system units, respectively [16,17]. Notably, subsequent articles by Mariano et al. (2021) and Xu et al. (2021) showcase the DEA’s application on varying scales—from Brazilian states, comparable DMU sets, Indian states and UTs, to U.S. states [18,19,20,21].

The spectrum of DMU definitions elucidated in these articles accentuates the adaptability of the DEA methodology. Whether evaluating healthcare systems, global models, specific nations, or even granular entities like hospitals, the DEA’s flexibility shines through. The chosen DMU inherently reflects the research’s goals, objectives, and contextual nuances, emphasizing the methodological richness and the expansive potential of DEA in diverse scientific pursuits.

### 3.4. Incorporation of Undesirable Outputs in DEA Amidst Pandemics

In the scrutinized compendium of articles, the conceptual shift towards embracing undesirable outputs is palpable, underscoring a recalibration of efficiency analytics in the face of global health adversities. Breitenbach et al. (2021) manifestly pave the way by accentuating input minimization, wherein undesirable outputs—particularly death and infection rates—are central to their DEA assessment of countries’ healthcare systems in their pandemic responses [10]. This augmentation of conventional DEA paradigms by spotlighting negative indicators represents a heightened sensitivity to the human cost and the complexities introduced by the pandemic. In a similar vein, Klumpp et al. (2022) align their methodological approach with an output-oriented ratio form under constant returns to scale (CRS), where efficiency is directly contingent upon proximity to an established efficiency frontier, implicitly factoring in the consequences of undesirable outcomes [11]. Mohanta et al. (2021) further enrich this discourse by employing the output-oriented BCC model, categorizing outputs into both desirable and undesirable, thus facilitating a nuanced bifurcation of outcomes, vital for a comprehensive efficiency ranking of Indian states and UTs [20].

Furthermore, Pereira et al. (2022) employ a network DEA model which, while focusing on stages like population, contagion, and hospitalization, inherently encompasses the repercussions of undesirable outcomes in their country-wide pandemic response analysis [16]. Adabavazeh et al. (2020) cement this narrative by defining their DMUs as health system units of 71 countries, with evaluation indices prominently comprising “Total Deaths” alongside other socio-economic indicators [17]. Their BCC output-based model seeks to pinpoint the highest efficiency ratio, further underscoring the integration of such undesirable metrics in their assessment. In contrast, Mariano et al. (2021) and Xu et al. (2021) implicitly account for these negative outputs by integrating the number of deaths and cases, respectively, in their DEA evaluations [18,19,20,21]. The curated articles manifestly illuminate the escalating importance of undesirable outputs in DEA, marking a pivotal paradigmatic shift in efficiency analyses, particularly in the face of unprecedented global crises.

### 3.5. Emphasis on External and Non-Discretionary Factors in DEA Analyses

From the curated articles, a nuanced pattern emerges, underlining the significance of external and non-discretionary factors in the context of DEA assessments. Md Hamzah, Yu, and See (2021) stand as a vanguard in this domain, encapsulating the essence by introducing non-discretionary inputs explicitly shaped by external dynamics [19]. Their model, which embraces these non-discretionary inputs, highlights the susceptibility of Decision Making Units (DMUs) to broader environmental and external factors. However, the permeation of such a perspective is not confined to just this singular article. Breitenbach et al. (2021), for instance, gravitates towards input minimization when underscoring undesirable outputs such as death and infection rates—elements inherently influenced by extraneous variables [10]. Similarly, the study by Klumpp et al. (2022) orbits around countries’ responses to the COVID-19 pandemic, subtly implying the role of global interconnections and external epidemiological pressures in determining efficiency frontiers [11]. In the work of Ordu et al. (2021), although DMUs are varied, ranging from hospitals to nations, the overarching efficiency assessments, especially when juxtaposed against the CCR and BCC models, tacitly acknowledge the external forces at play [15]. Pereira et al.’s (2022) exploration of the multi-stage pandemic response across fifty-five countries intrinsically factors in external dynamics, from global contagion trends to international health advisories, influencing each country’s response trajectory [16].

Moreover, Mohanta et al. (2021) dive into the realm of states and Union Territories of India, inherently suggesting the non-discretionary nature of federal policies and directives that bear upon regional healthcare systems [20]. Lupu and Tiganasu’s (2022) emphasis on European nations during 2020 echoes the inescapable clutches of global dynamics and European Union policies intricately affecting each nation’s pandemic response [13]. Kuzior et al. (2022) dissect healthcare system models, inadvertently acknowledging the historical, political, and socio-economic forces that sculpted these systems over time [12]. Xu et al.’s (2021) incorporation of machine learning frameworks to study U.S. states’ efficiencies beckons consideration of a myriad of environmental variables as predictors, reinforcing the interplay of external factors [21]. Collectively, these articles underscore the indispensable need for recognizing and integrating non-discretionary and external elements in DEA evaluations, elucidating a holistic, interconnected, and multi-faceted perspective on efficiency dynamics.

### 3.6. Integration of Modern Technologies in DEA Analyses

A perspicacious observation across the compendium of articles subjected to this scoping review delineates an intriguing confluence: the nexus between traditional DEA techniques and burgeoning computational methodologies. Xu et al. (2021) stand at the forefront of this innovative amalgamation, intertwining DEA with machine learning (ML) paradigms. In their elucidation, U.S. states’ efficiencies, as gauged during the COVID-19 pandemic, were not merely passively assessed. Instead, DEA-derived efficiency scores became pivotal target variables within advanced ML frameworks. Harnessing a suite of 15 environmental variables as predictors, their approach manifested an interdisciplinary synergy, magnifying the analytical depth of DEA through the predictive prowess of ML [21].

The profundity of Xu et al.’s (2021) integration is hardly an isolated exemplar within the academic milieu [22]. While their work remains a paragon, several articles, albeit with less overt technological integration, exhibit subtle nuances of modern technology interfusion. For instance, Azadi et al. (2022) showcased adeptness in maneuvering a plethora of data types concurrently, a capability hinting at sophisticated data processing tools often aligned with contemporary computational sciences [9]. Similarly, Klumpp et al.’s (2022) employment of DEA window analysis and Network DEA resonates with the advanced algorithmic strategies burgeoning in the modern era. In summation, this synthesis underscores an epoch where the sanctified realms of traditional efficiency analyses are harmoniously converging with the vanguard of technological evolution, heralding an era of enriched academic profundity [11].

## 4. Discussion

The articles examined in this scoping review provided valuable insights into the intricacies of DEA Efficiency Analysis, particularly in the health sector in light of the COVID-19 pandemic. The subsequent discussion aims to juxtapose our findings with extant literature, while highlighting nuances and insights in the realm of DEA.

### 4.1. Selective Variation of DMU Definition

A principal observation in the current review is the diverse assortment of DMU definitions employed across various health systems’ analyses. As observed in seminal works, such as Charneset al. (2000), DEA has been celebrated for its ability to account for the multi-faceted nature of DMUs, where the unit of analysis can range from institutions like hospitals to nations. Our findings are consonant with this assertion [1]. The present exploration demonstrated the malleability of DEA, as it is implemented to assess everything from healthcare supply chains to entire country responses. This aligns with Avkiran (2001), who emphasized the importance of a flexible DMU definition based on the specific objectives and context of each research endeavor [23]. It was further substantiated by the application of DEA in different scales, accentuating its adaptability, as elucidated by Mariano et al. (2021) and Xu et al. (2021) [18,19,20,21].

### 4.2. Undesirable Outputs in DEA Analysis

Incorporating undesirable outputs in DEA amidst a global crisis represents a significant advancement in efficiency analyses. Prior to the COVID-19 pandemic, DEA literature primarily emphasized operational efficiency in health systems without centralizing undesirable outputs such as mortality rates [24]. However, the present review illuminated a noticeable shift in this narrative, as seen in studies by Breitenbach et al. (2021) and Klumpp et al. (2022) [10,11,12], aligning with Emrouznejad and Yang’s (2018) assertions of the need for an evolved DEA that captures the nuances of contemporary challenges [4]. Recognizing these negative outcomes is not just methodologically novel but also ethically imperative, especially in light of global health adversities.

### 4.3. Emphasis on External and Non-Discretionary Factors

The articles surveyed underscored the inextricable influence of external and non-discretionary factors on efficiency assessments. The external dynamic, as evidenced in our review, resonates with Cook and Seiford’s (2009) contention that real-world scenarios, especially crises, render DMUs susceptible to environmental pressures [22]. While DEA has traditionally prided itself on isolating managerial efficiency [2], the advent of global challenges like the pandemic necessitates a broader, more comprehensive analysis. Our findings corroborate this need, highlighting the indispensability of accounting for these overarching factors in DEA appraisals.

### 4.4. Integration of Modern Technologies

The entwining of traditional DEA techniques with burgeoning computational methodologies emerges as a groundbreaking trend. This aligns with the assertions of Tone and Tsutsui (2014), who highlighted the expanding horizons of DEA through technological advancements [7]. The pioneering work of Xu et al. (2021) exemplifies this shift, showcasing the immense potential of coupling DEA with advanced methodologies like ML [21]. It underscores an era where efficiency analysis is not static but dynamically evolving with technological progressions.

The adaptation and evolution of Data Envelopment Analysis (DEA) methodologies, particularly in the context of healthcare efficiency analysis during the COVID-19 pandemic, are well-documented in the current scoping review. Consistent with previous literature, DEA’s inherent flexibility is effectively harnessed to address pandemic-specific challenges, such as the integration of undesirable outputs and the influence of external factors [22]. Notably, the inclusion of mortality rates as undesirable outputs in DEA models marks a significant departure from traditional applications, which have predominantly focused on operational efficiencies [24]. This shift not only aligns with but also extends previous academic recommendations for more holistic approaches in efficiency assessments, emphasizing the necessity to capture broader societal impacts and externalities [5].

Furthermore, the integration of advanced computational methods, including machine learning, into DEA frameworks represents a progressive alignment with contemporary analytical needs. This integration enhances DEA’s analytical capabilities, enabling more nuanced and dynamic efficiency evaluations that are crucial under the rapidly changing conditions of a global health crisis. The application of DEA across diverse geographical and operational settings reaffirms its robustness and adaptability, consistent with its documented applications across various sectors and regions [6].

The reviewed studies not only corroborate the established versatility of DEA but also significantly enrich its methodological framework to better address the complexities introduced by the COVID-19 pandemic. These advancements provide valuable insights for future research and underscore the potential of DEA in navigating the operational challenges posed by global health emergencies.

## 5. Conclusions

This scoping review’s findings, in essence, not only illuminate the evolving paradigms of DEA application in health systems but also beckon a reconsideration of conventional methodologies. As we confront unprecedented global challenges, it becomes pivotal for scholarly discourses to resonate with contemporary demands, ensuring both methodological rigor and contextual relevance. The synchrony between traditional efficiency analyses and cutting-edge methodologies heralds a promising trajectory for future research endeavors.

## Figures and Tables

**Table 1:** Characteristics of the articles reviewed, created by author.

Author	Year	Key Methodology/Technique	Main Focus/Contribution	Evolution and Diversification	Temporal Dynamics	Selective Variation of DMU	Incorporation of Undesirable Outputs	Emphasis on External and Non-Discretionary Factors	Integration of Modern Technologies
Azadi et al. [9]	2022	Network RDM model	Healthcare supply chains during pandemics	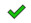		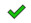			
Breitenbach et al. [10]	2021	VRS approach	Healthcare systems of countries during pandemics	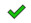		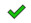	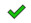	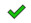	
Klumpp et al. [11]	2022	DEA model with window analysis	Time-series efficiency assessment	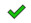	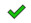	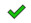	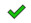		
Kuzior et al. [12]	2022	Multivariate exploratory techniques	Efficiency analysis	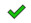		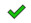			
Lupu and Tiganasu [13]	2022	Multisource data analysis	Dynamic efficiency evaluations of European nations	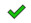		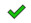		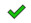	
Mourad et al. [14]	2021	Nonparametric mathematical programming with Tobit regression	Multi-model analytical paradigm	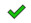					
Ordu et al. [15]	2021	CCR, BCC, and super efficiency DEA method	Enhanced traditional methodologies	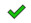	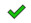	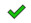		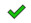	
Pereira et al. [16]	2022	Multi-stage systems with simulations	Balance between social welfare and resource optimization	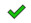	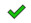	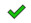	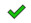	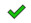	
Adabavazeh et al. [17]	2020	Diverse evaluation indices with BCC output-based model	Harmonized analytical paradigm across 71 countries	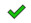		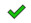	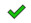		
Mariano et al. [18]	2021	Network DEA	Regional disparities in health system efficiencies	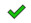		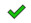			
Md Hamzah, Yu, and See [19]	2021	Three-stage NDEA model	Resource utilization dynamics	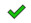				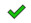	
Mohanta et al. [20]	2021	BCC model with ‘Maximal Balance Index’	Pandemic management in India	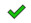		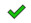	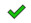	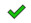	
Xu et al. [21]	2021	DEA merged with machine learning frameworks	Comprehensive efficiency analysis of U.S. states during the pandemic	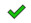	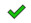		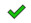		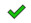

Green checkmark denotes that the reviewed work is featured in the respective category.
